# The efficacy of aprepitant for the prevention of postoperative nausea and vomiting: A meta-analysis

**DOI:** 10.1097/MD.0000000000034385

**Published:** 2023-07-21

**Authors:** Yingchao Liu, Xinli Chen, Xiaohua Wang, Huohu Zhong, Hefan He, Yibin Liu, Yuewen Liao, Zhigang Pan, Weipeng Hu, Weifeng Liu, Feng Zheng

**Affiliations:** a Department of Anesthesiology, the Second Affiliated Hospital of Fujian Medical University, Quanzhou, China; b Department of Ultrasound, the Second Affiliated Hospital of Fujian Medical University, Quanzhou, China; c Department of Neurosurgery, the Second Affiliated Hospital, Fujian Medical University, Quanzhou, China.

## Abstract

Postoperative nausea and vomiting (PONV) is one of the common adverse reactions after surgery. Recent randomized controlled trials (RCTs) investigating antiemetic drugs suggest that aprepitant has the strongest antiemetic effect of any single drug. This meta-analysis aimed to explore the efficacy of aprepitant for preventing PONV based on the existing literature.

**Methods:**

To identify RCTs investigating the use of aprepitant for PONV prevention, we searched PubMed, Embase, and Cochrane Library databases for articles published prior to March 20, 2022. Seventeen RCTs were identified, with 3299 patients, meeting the inclusion criteria. PONV incidence, complete response, 80 mg aprepitant combined with dexamethasone and ondansetron, vomiting, nausea, and analgesic dose-response were the main outcomes measured.

**Results:**

Compared with the control group, PONV incidence was significantly reduced among those receiving aprepitant (odds ratio [OR]: 0.34; 95% confidence interval [CI]: 0.26, 0.44; *P* < .0001), with a more complete response (OR: 1.35; 95% CI: 1.14, 1.59; *P* = .0004). Supplementation of 80 mg aprepitant in combination with dexamethasone and ondansetron substantially improved the effects of PONV (OR: 0.36; 95% CI: 0.16, 0.82; *P* = .01). Further, administration of 80 mg aprepitant was better at preventing vomiting than nausea (OR: 8.6; 95% CI: 3.84, 19. 29; *P* < .00001). No statistically significant difference between the dose-response of analgesics was identified (mean difference: −1.09; 95% CI: −6.48, 4.30; *P* = .69). The risk of bias was assessed independently by paired evaluators.

**Conclusion:**

Aprepitant effectively reduces the incidence of PONV; however, the effects of postoperative analgesia require further exploration.

## 1. Introduction

Postoperative nausea and vomiting (PONV) refers to nausea, vomiting, or retching in the post-anesthesia care unit during the 24-hour period following surgery.^[1]^ As one of the most common postoperative adverse reactions, reported to be even more unbearable than pain, PONV not only increases the risk of postoperative complications but also brings discomfort to patients, reduces patient satisfaction, and prolongs hospital stays.^[2–4]^ It is also one of the main reasons for unplanned readmission, leading to a great burden on patients.^[5,6]^ The incidence of PONV is approximately 30% among the general population and 80% among the high-risk population, with an Apfel risk score of 3 or 4.^[7]^ At present, the first-line drug for preventing PONV is the 5-HT3 receptor antagonist ondansetron.^[8]^ Although such antiemetics are widely used, PONV prevention remains challenging in postoperative care.^[2]^ As anesthesia methods have advanced, an increasing number of strategies for PONV prevention have emerged. Updated antiemetic drugs and prevention programs have been introduced, which may improve safety, extend the duration of drug effectiveness, and enhance efficacy.^[2,9,10]^ A recent consensus regarding PONV prevention in high-risk patients suggested that a multi-mode prevention strategy should be considered, combining antiemetic drugs with different mechanisms.^[3]^ Studies have shown that the efficacy of combined prevention methods depends on the intensity of a single drug.^[1,11]^ In a recent meta-analysis examining the effectiveness of antiemetic drugs, a neurokinin-1 (NK-1) receptor antagonist was considered the most effective and highest-quality single drug for PONV prevention. Further, its effectiveness was similar to that of dexamethasone-ondansetron combination therapy.^[11]^ Currently, aprepitant is the most used NK-1 receptor antagonist. Although an increasing number of studies on the use of aprepitant in clinical practice have been reported in recent years, its effectiveness in preventing PONV remains unclear.^[4,12–15]^ We conducted a meta-analysis of randomized controlled trials (RCTs) to understand better how aprepitant use affects current PONV management strategies with a view to improving patient postoperative comfort.

## 2. Methods

### 2.1. Study inclusion and exclusion criteria

Studies meeting the following criteria were included in the present meta-analysis: RCTs, those assessing adults > 18 years with an American Society of Anesthesiologists score of I–III, those with patients who underwent elective surgeries after using antiemetics in the preoperative period, those with an intervention group that received aprepitant, and those that administered traditional antiemetic drugs to the control group. Studies that included patients receiving preoperative or preventive antiemetic drugs were excluded. Reviews, case reports, and studies published in languages other than English were excluded. No informed consent from patients was required in the present study.

### 2.2. Search strategy and study selection

Our meta-analysis adheres to PRISMA guidelines.^[16]^ Two reviewers (Y.L. and W.L.) independently searched the PubMed, Embase, and Cochrane Library databases for articles published before March 20, 2022. The search strategy included the following combinations of mesh terms and free words: “Neurokinin-1 Receptor antagonists” and “PONV” (including “postoperative nausea and vomiting,” “postoperative emesis,” and “postoperative vomiting”). After retrieval, both reviewers independently determined which articles should be included according to predefined inclusion and exclusion criteria. Any discrepancies that occurred during the study selection process were resolved by a senior author (F.Z.).

### 2.3. Data collection and outcome assessment

Two reviewers (Y.L. and W.L.) independently extracted data using a predesigned, standardized table. Data assessed included the following: first author, publication year, country, study design, surgery type, anesthesia, the dose of aprepitant, intervention group, and comparator. The main outcome assessed was the incidence of PONV within 24 hours postoperatively. Secondary outcomes considered included the following: complete response (CR, no vomiting, and no need for any antiemetic or rescue drugs), use of rescue antiemetic drugs, the incidence of nausea or vomiting within 24 hours of surgery, and use of postoperative analgesic drugs.

Dichotomous data regarding those with or without PONV were converted to incidence. The mean and standard deviation was used to record continuous data. When the data were incomplete, we attempted to email the corresponding author to obtain detailed information. If the authors did not reply, available data were extracted.

### 2.4. Risk of bias assessment

The quality of included studies was evaluated using the bias risk assessment tool provided by the Cochrane Collaboration Network. Sequence generation, allocation sequence concealment, blinding, incomplete outcome data, selective outcome reporting, and other potential sources of bias were also assessed. Based on the assessment, the risk of bias was set as low, high, or unclear (indicating a lack of information or uncertainty regarding potential bias).^[17]^

### 2.5. Synthesis methods and effect measures

We assessed dichotomous outcomes and estimated treatment effectiveness via a pairwise comparison that used odds ratios (ORs) and 95% confidence intervals (CIs). This meta-analysis used Review Manager software (version 5.4; The Cochrane Collaboration, 2020; Nordic Cochrane Center, Copenhagen, Denmark). Two evaluators independently completed the quality assessment. *I*^2^ statistics were used to measure statistical heterogeneity. Since the treatment results varied according to regional differences and varying research characteristics, a random effects model was employed. According to recommendations of the Cochrane Statistical Methods Group, the significance level of the *P* value used to assess heterogeneity was set to 0.1. Further, the *I*^2^ statistic indicated low, moderate, substantial, and considerable heterogeneity when values were 0% to 40%, 30% to 60%, 50% to 90%, and 75% to 100%, respectively.^[17]^ Statistically significant heterogeneity was indicated at *P* < .10 and *I*^2^ > 50%. Studies that contributed most to heterogeneity were excluded from sensitivity analysis. The analysis findings were used to determine the robustness of the results.

## 3. Results

### 3.1. Study selection

**Figure 1.**
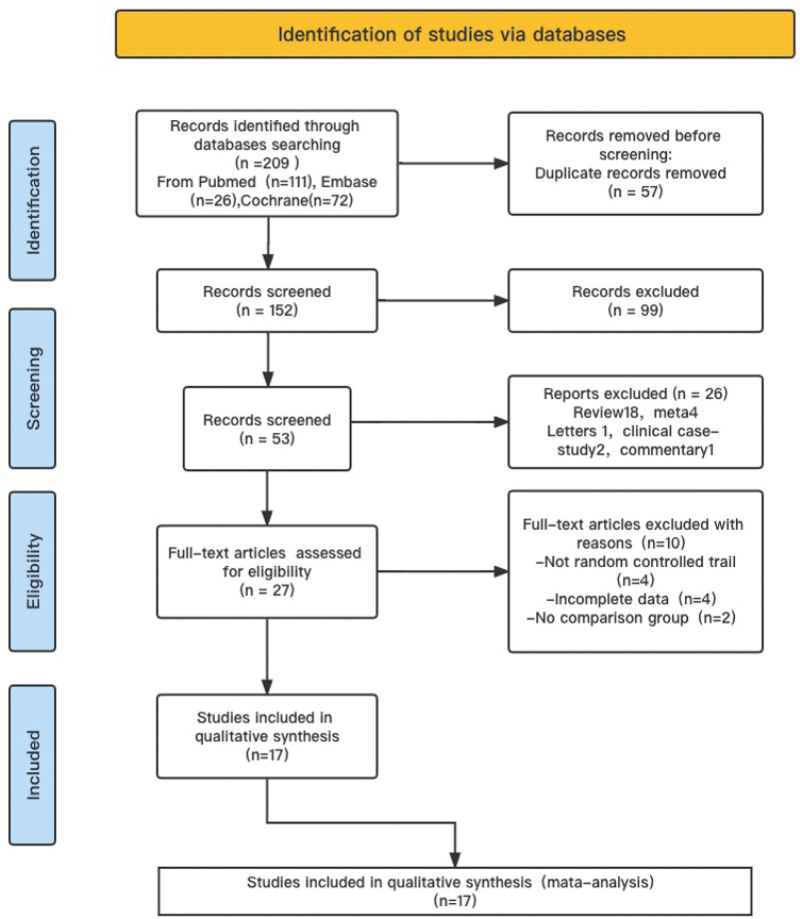
The flow chart of the search algorithm.

Preliminary retrieval resulted in the identification of 209 articles from the databases considered. After initial screening, 17 studies involving 3299 participants were included in the analysis (Fig. 1). Among the 17 studies, a trial by Ham et al^[9]^ was published as a summary. Since the work reported key outcome indicators, it was included in the present analysis.

### 3.2. Study characteristics

Characteristics of all 17 included studies are listed in Table 1. All studies considered were RCTs. Types of surgeries were as follows: 10 gynaecological operations (1 ambulatory, 4 open, and 5 laparoscopic), 2 orthopedic operations, 2 weight loss operations, and 2 neurosurgery operations. All operations were performed under general anesthesia (including 5 studies that used fentanyl for postoperative patient-controlled analgesia). The following different doses of aprepitant were used in 17 trials: 40 mg,^[22,25,27–29]^ 80 mg,^[9,12–15,18–21,23,26]^ and 125 mg.^[20,21,23,27–29]^ In the intervention group, a precipitant was primarily or adjunctively used as an emetic agent. In the control group, ondansetron, dexamethasone, palonosetron, ramosetron, or placebo was administered alone or in combination. A placebo was used as the control group in 2 studies.^[23,26]^

**Table 1:** Characteristics of studies included.

Name and publication year	Country	Study design	Surgery type	Anesthesia	Dose of aprepitant	Intervention	Comparator	Adverse events with aprepitant
Thanuja^[18]^ 2021	India	RCT	Day care gynecologic laparoscopy	VICA	80 mg	Dex, Ondan, Apre	Dex, Ondan	Pain, agitation, and lethargy (2 groups did not differ significantly)
Grigio^[14]^ 2020	Brazil	Double-blinded RCT	Unilateral mastectomy	TIVA	80 mg	Dex, Palono, Apre	Dex,Palono	N/A
Spaniolas^[15]^ 2020	US	RCT	Sleeve gastrectomy	TIVA	80 mg	Dex, Ondan, Apre	Dex, Ondan	N/A
Yoo^[12]^ 2018	Korea	Single-blinded RCT	Elective surgery (Major orthopedic operation)	VICA IV-PCA	80 mg	Apre, Palono	Palono	Dizziness/headache
De Morais^[13]^ 2018	Brazil	Double-blinded RCT	laparoscopic surgeries	TIVA	80 mg	Multimodal regimen and Apre	Multimodal regimen	N/A
Ham^[9]^ 2016	Korea	Double-blinded RCT	Laparoscopic gynecologic surgery	VICA IV-PCA	80 mg	Apre, Ondan	Ondan	N/A
Kawano^[19]^ 2015	Japan	Double-blinded RCT	Elective arthroplasty	VICA	80 mg	Apre	Dex	Dizziness/headache
Sinha^[20]^ 2013	US	Double-blinded RCT	Bariatric surgery	VICA	80 mg 125 mg	Apre Ondansetron	Ondan	N/A
Lim^[21]^ 2013	Korea	RCT	N/A	N/A	80 mg 125 mg	Apre, Ondan	Ondan	No evident side effect of aprepitant was observed.
Bergese^[22]^ 2012	US	Double-blinded RCT	Neurosurgery	GA	40 mg	Dex, Prome, Apre	Dex, Prome, Ondan	Cerebrovascular hematoma in 2 patients in aprepitant (likely unrelated to drug)
Seon Jung^[23]^ 2012	Korea	Double-blinded RCT	Laparoscopic gynecologic surgery	VICA IV-PCA	80 mg 125 mg	Apre	Placebo	Dose-related dizziness (3 patients in 125 mg, 1 patient in 80 mg), headache, dyspepsia, abdominal distension
Lee^[24]^ 2012	Korea	RCT	Gynecological surgery	VICA IV-PCA	80 mg	Apre, Ramo	Ramo	Dizziness/headache/sedation (The incidence was higher in the ramosetron group)
Habib^[25]^ 2011	US	Double-blinded RCT	Craniotomy	VICA	40 mg	Apre, Dex	Ondan, Dex	N/A
Kakuta^[26]^ 2011	Japan	RCT	Gynecological surgery	VICA	80 mg	Apre	Placebo	N/A
Diemunsch^[27]^ 2007	France	Double-blinded RCT	Open abdominal surgery	VICA	40 mg 125 mg	Apret	Ondan	Constipation/headache/Bradycardia
Diemunsch^[28]^ 2007	France	Double-blinded RCT	Open abdominal surgery	VICA	40 mg 125 mg	Apre	Ondan	N/A
Gan^[29]^ 2007	US	Double-blinded RCT	Open abdominal surgery gynecologic	VICA	40 mg 125 mg	Apre	Ondan	Mild constipation, respiratory depression, sedation

Apre = aprepitant, Dex = dexamethasone, GA = general anesthesia, IV-PCA = intra-venous patient-controlled analgesia, N/A = not available, Ondan = ondansetron, Prome = promethazin, Ramo = ramosetron, TICA = total intravenous anesthesia, VICA = vein inspiration compound anesthesia.

### 3.3. Analysis of study quality

**Figure 2.**
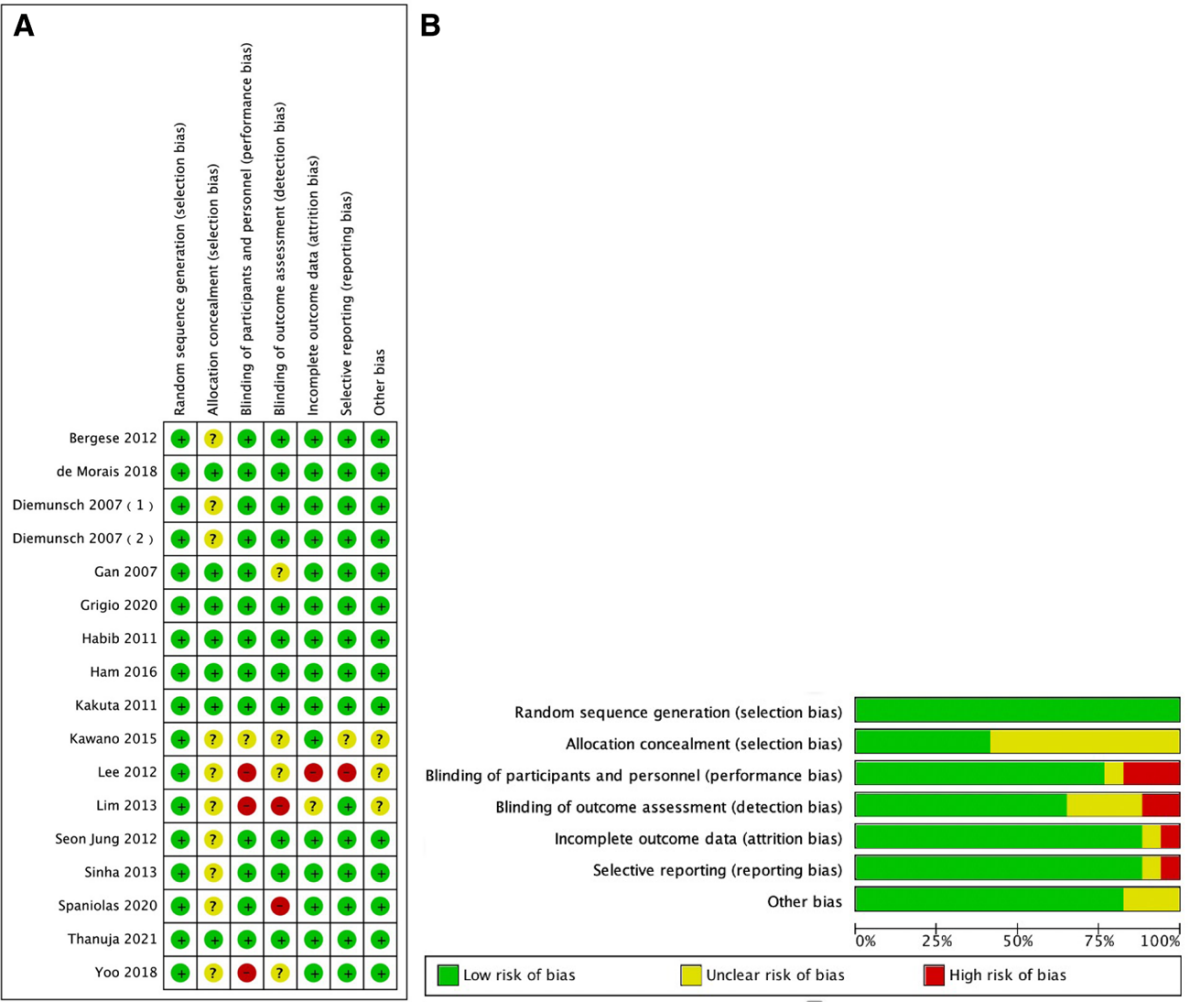
Quality analysis of all 17 studies included in the meta-analysis. The risk of bias is summarized (A) and graphed (B).

We evaluated the eligibility criteria of the 17 identified studies using the Cochrane collaboration tool.^[17]^ The quality analysis of all 17 studies is shown in Figure 2.

### 3.4. Results of individual studies

#### 3.4.1. Incidence of PONV.

**Figure 3.**
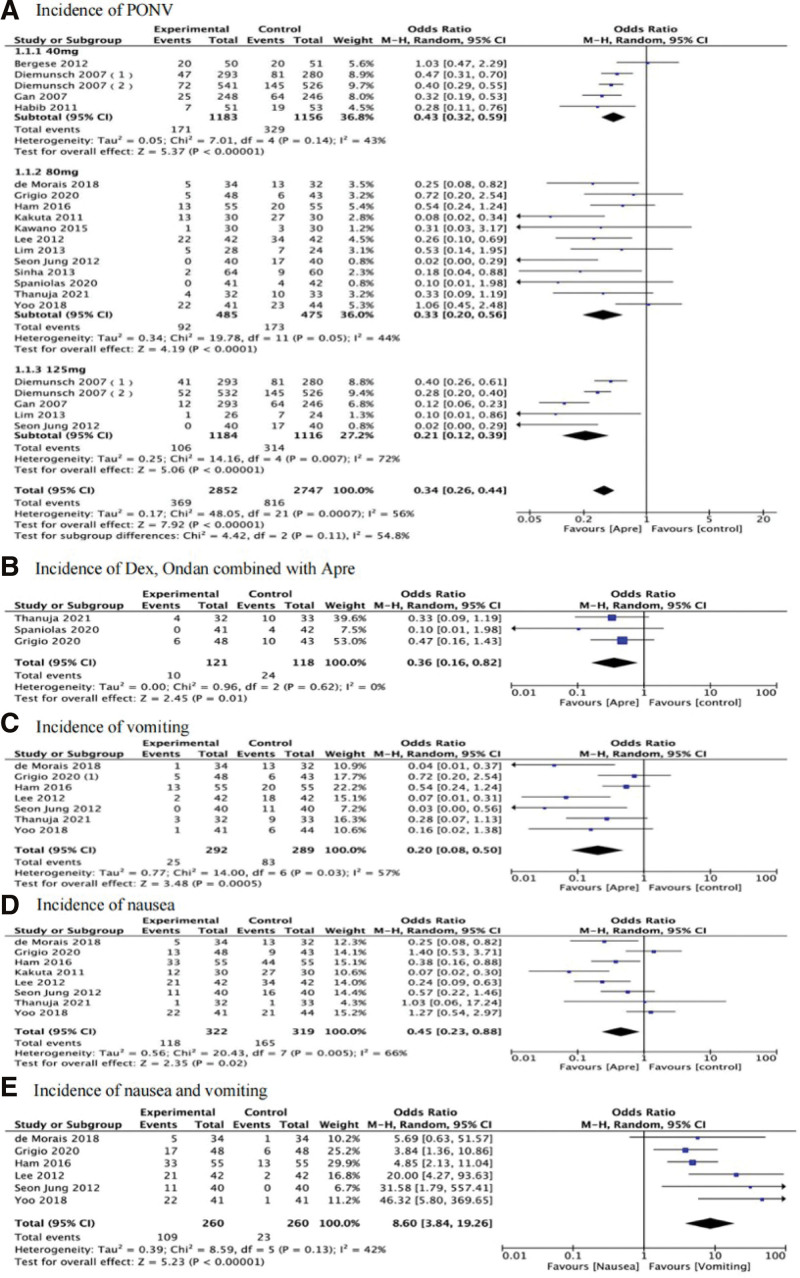
Pooled data of the included studies. CI = confidence intervals, PONV = postoperative nausea and vomiting.

Pooled results of the 17 studies showed that the incidence of PONV of the aprepitant group (OR: 0.34, 95% CI: 0.26, 0.44, *P* < .05) was significantly lower than that of the control group (Fig. 3A). Owing to significant heterogeneity (*I*^2^ = 56%), a sensitivity analysis was employed to assess the robustness of the findings. After excluding 1 trial by Gan et al^[29]^ heterogeneity was significantly reduced (*I*^2^ = 45%); however, findings remained significant (OR: 0.37, 95% CI: 0.28, 0.49, *P* < .00001) (Figure S1A, Supplemental Digital Content, http://links.lww.com/MD/J322).

Furthermore, pooled data from 3 studies that analyzed dexamethasone and ondansetron combined with aprepitant^[14,15,18]^ showed that the incidence of PONV in dexamethasone and ondansetron combined with aprepitant groups was significantly lower than that of control groups given dexamethasone combined with ondansetron (OR: 0.36, 95% CI: 0.16, 0.82, *P* < .05) (Fig. 3B).

#### 3.4.2. Incidence of vomiting.

Seven studies reported the incidence of vomiting.^[9,12–14,18,23,24]^ A significant advantage was detected in the group treated with aprepitant (OR: 0.20, 95% CI: 0.08, 0.50, *P* = .0005) (Fig. 3C). Owing to significant heterogeneity (*I*^2^ = 57%), a sensitivity analysis was employed to assess the robustness of the findings. After the exclusion of 1 trial by Ham et al^[9]^ findings remained significant (OR: 0.15, 95% CI: 0.05, 0.42, *P* = .0003) (Figure S1B, Supplemental Digital Content, http://links.lww.com/MD/J322).

#### 3.4.3. Incidence of nausea.

In 8 studies analyzing the incidence of nausea,^[9,12–14,18,23,24,26]^ pooled data indicated that the incidence of nausea was significantly lower in the intervention than in the control group (OR: 0.45; 95% CI: 0.23, 0.88; *P* = .02; *I*^2^ = 66%) (Fig. 3D). Owing to the existence of significant heterogeneity (*I*^2^ = 56%), a sensitivity analysis was employed to assess the robustness of the findings. After the exclusion of 1 trial by Kakuta et al^[26]^ the finding remained significant (OR: 0.56; 95% CI: 0.31, 1.00; *P* = .05) (Figure S1C, Supplemental Digital Content, http://links.lww.com/MD/J322).

#### 3.4.4. Comparison of the incidence of nausea versus vomiting.

Six studies reported the incidence of nausea and vomiting.^[9,12–14,23,24]^ Findings showed that administration of 80 mg aprepitant reduced postoperative vomiting significantly more than postoperative nausea (OR: 8.6; 95% CI: 3.84, 19.29, *P* < 0. 00001; *I*^2^ = 42%) (Fig. 3E).

#### 3.4.5. CR.

Eight studies reported the incidence of CR.^[9,20,22,23,25,27–29]^ The CR rate of the aprepitant group was significantly higher than that of the control group (OR: 1.35; 95% CI: 1.14, 1.59; *P* = .0004). Substantial heterogeneity was not observed (*I*^2^ = 36%) (Figure S1D, Supplemental Digital Content, http://links.lww.com/MD/J322).

#### 3.4.6. Use of a rescue antiemetic.

An assessment of 12 studies that assessed the use of a rescue antiemetic^[9,12–14,18,20,21,24–29]^ showed no significant inter-group differences (OR: 0.86; 95% CI: 0.68,1. 09; *P* = .21) (Figure S1E, Supplemental Digital Content, http://links.lww.com/MD/J322). Subgroup analyses were performed based on the dosage of the aprepitant administered. This assessment examined the use of rescue drugs among 40 mg aprepitant and control groups.^[25,27,29]^ Pooled data revealed no significant difference between the intervention (40 mg aprepitant) and control groups (OR: 1.09; 95% CI: 0.86, 1.38; *P* = 0. 48). The incidence of rescue drug use in the 80 mg aprepitant group was significantly lower than that in the control group (OR = 0.58, 95% CI: 0.37, 0.91; *P* = .02). No significant difference was detected when the 125 mg aprepitant and control groups were compared (OR: 0.94; 95% CI: 0.61, 1.46; *P* = .79).

#### 3.4.7. Use of postoperative analgesics.

Two studies recorded the use of postoperative analgesics after 80 mg aprepitant administration.^[14,26]^ No significant difference between the 80 mg aprepitant and control groups was observed (mean difference: −1.09; 95% CI: −6.48, 4.30; *P* = .69) (Figure S1F, Supplemental Digital Content, http://links.lww.com/MD/J322).

### 3.5. Risk of bias

Publication bias data is presented using funnel plots (Figure S2, Supplemental Digital Content, http://links.lww.com/MD/J323). Bias was examined using Egger’s test (Table 2). No significant publication bias was observed in this study.

**Table 2:** Publication bias assessment of this meta-analysis.

Std_Eff	Coefficient	Std. err.	T	*P*> t	[95% conf. interval]	
Slope	2014.95	.6642029	3033.64	.000	2013.515	2016.385
Bias	.0244783	.2081843	0.12	.908	−.4252765	.4742332

Std.err = standard error, Std_Eff = standard effect.

## 4. Discussion

**Figure 4.**
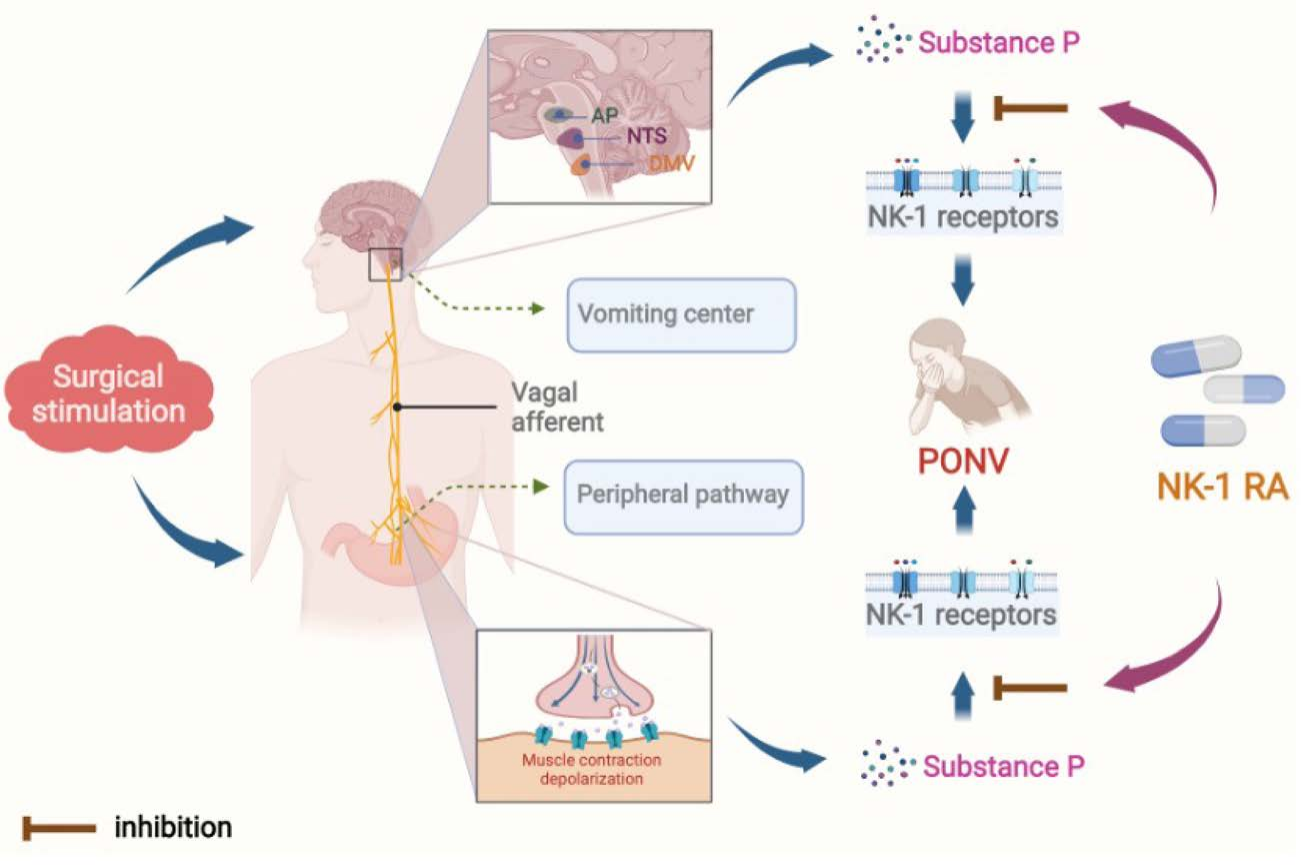
When the patient is stimulated surgically, substance P is produced via central and peripheral pathways. Binding of the substance to the NK-1 receptor leads to PONV. An Nk-1 receptor antagonist blocks binding of substance P to the NK-1 receptor, which can reduce the incidence of PONV. AP = area postrema, DMV = dorsal motor nucleus of vague nerve, NK-1 RA = K-1 receptor antagonist, NTS = nucleus tractus solitarily, PONV = postoperative nausea and vomiting.

Although the physiological pathway of PONV has not been fully elucidated, some nerve receptors, including dopamine, 5-HT3, and NK-1, have been identified as key components involved in regulating the process of nausea and vomiting.^[30,31]^ The NK-1 receptor belongs to the tachykinin family of G-proteins, for which substance P is a ligand.^[32]^ Studies have shown that low doses of intravenously injected substance P causes vomiting in dogs, and high doses of intraperitoneally injected substance P causes vomiting in hamsters.^[33,34]^ Further, NK-1 receptor agonists have been shown to cause vomiting in hamsters in a dose-dependent manner. The NK-1 receptor and substance P are expressed in central and peripheral vomiting reflexes in the area postrema, nucleus tractus solitarius (NTS), vague afferent nerve, gastrointestinal neurons, and dorsal motor nucleus of the vague nerve.^[32,35]^ When the NK-1 receptor binds and is activated by endogenous substance P, a signal is transmitted to the NTS, which projects to the vomiting center of the local brainstem area, resulting in a vomiting reflex and the middle and forebrain, causing nausea.^[36]^ NK-1 receptor antagonists block the NK-1 receptor from binding substance P. This process affects the final common vomiting pathway that includes the NTS and dorsal motor nucleus of the vague nerve, producing an antiemetic effect (Fig. 4 describes the functioning of NK-1 receptor antagonists).^[37,38]^

Results of our meta-analysis showed that the prophylactic use of aprepitant (40, 80, and 125 mg) alone or in combination with ondansetron, dexamethasone, palonosetron, ramosetron, and placebo in patients with high-risk factors significantly reduces the incidence of PONV and increases the CR rate. Moreover, aprepitant reduces the use of rescue drugs, although results were not statistically significant. This lack of significance may have been due to the subjective expression of patients.^[39]^ These findings are consistent with the previous meta-analysis by Weibel et al, Liu et al and Singh et al^[11,40,41]^ Compared with previous meta-analyses, this study further explored the specific efficacy of aprepitant in preventing PONV and its role in multimodal antiemesis. Five new outcome indexes were added, including the incidence of nausea, the incidence of vomiting, the incidence of differential comparison between nausea and vomiting, the incidence of combined medication and the correlation with the dosage of analgesic drugs. Dexamethasone, ondansetron, and aprepitant have been recommended to prevent chemotherapy-induced nausea and vomiting.^[42]^ Results of our analysis show that compared with the classic combination of dexamethasone and ondansetron, a combination of aprepitant, dexamethasone, and ondansetron effectively reduces the incidence of PONV, and no significant side effects occurred. This finding could provide a new direction for PONV guidelines recommending that high-risk patients should combine 3 or 4 antiemetic prophylaxes with baseline risk reduction strategies.

Additionally, our results showed that aprepitant was effective in reducing the incidence of nausea, as well as in reducing the incidence of vomiting, and aprepitant was more effective in preventing vomiting than nausea, suggesting that aprepitant was a potent antiemetic. This is consistent with the ideas presented by previous authors.^[9,26]^ This effect may be related to the drug mainly affecting the vomiting center, while a mostly peripheral mechanism underlies nausea. In the studies included in the present analysis, aprepitant was administered orally 1 to 3 hours before surgery. Most trials have found that aprepitant effectively delays postoperative nausea and vomiting. This may also be due to the longer half-life and increased duration of activity of aprepitant versus other 5-HT3 receptor antagonists, including ondansetron.^[20,21,24]^ Both characteristics of aprepitant confer a significant therapeutic advantage.

The use of opioids during and after surgery is one of the main causes of PONV.^[43]^ Kakuta et al^[26]^ revealed that when aprepitant was used to prevent PONV, opioid dosages were also reduced. Considering the role of substance P in pain, NK-1 antagonists may also have analgesic effects. However, the results of our meta-analysis showed that analgesic dosage was not significantly affected by the use of NK-1 receptor antagonists. This may be related to the lack of data in the studies we included. Further research investigating this issue is warranted.

Finally, it is worth mentioning that 5-HT3 antagonists and anti-dopamine drug use may lead to a prolonged QT interval, while anticholinergic drugs, antihistamine drugs, and dexamethasone may lead to delirium in high-risk patients.^[44,45]^ At present, there is no evidence that aprepitant has significant side effects.^[2,40]^ Therefore, aprepitant is a potential alternative to other drugs such as ondansetron, dexamethasone, romansetron, and palonosetron that have contraindications. Pooled results considered in the present analysis showed that aprepitant may better prevent vomiting than nausea and improve patient satisfaction, a finding that is consistent with those of previous studies.^[12–14]^

Our meta-analysis had some limitations. First, the studies considered included patients who underwent different anesthesia methods and surgeries. Further, there was a lack of data regarding opioid use and the duration of surgery, which likely increased between-study heterogeneity. In addition, the control group included patients given antiemetic drugs that functioned via different mechanisms. The different efficacies of these drugs may also have contributed to the heterogeneity of our results. Second, due to the lack of available data, no analysis of patient satisfaction, clinical outcomes beyond 24-hours (such as delayed discharge and readmission due to PONV), or cost-effectiveness was considered. A further study of these outcomes is warranted.

## 5. Conclusion

Our study revealed that aprepitant effectively reduces the incidence of PONV among high-risk patients. Further, vomiting was more significantly prevented by aprepitant than nausea. The use of dexamethasone and ondansetron combined with aprepitant may be most effective for preventing PONV. Future studies with larger sample sizes are warranted to confirm our findings.

## Author contributions

**Conceptualization:** Weifeng Liu.

**Data curation:** Xiaohua Wang.

**Formal analysis:** Huohu Zhong.

**Investigation:** Yuewen Liao, Zhigang Pan.

**Methodology:** Xinli Chen.

**Software:** Hefan He, Yibin Liu.

**Validation:** Weipeng Hu, Weifeng Liu.

**Writing – original draft:** Yingchao Liu.

**Writing – review & editing:** Feng Zheng.

## Supplementary Material
