# Supplementary material for: The efficacy of aprepitant for the prevention of postoperative nausea and vomiting: A meta-analysis
Source: Medicine (Baltimore). 2023 Jul 21;102(29):e34385. doi: 10.1097/MD.0000000000034385 (PMC10662847; doi:10.1097/MD.0000000000034385)

Figure S1. Pooled additional aggregate data included in the study.

A. Incidence of PONV after sensitivity analysis

Study or Subgroup	Experimental Events	Experimental Total	Control Events	Control Total	Weight	Odds Ratio M-H, Random, 95% CI
**1.1.1 40mg**						
Bergese 2012	20	50	20	51	5.8%	1.03 [0.47, 2.29]
Diemunsch 2007 (1)	47	293	81	280	10.5%	0.47 [0.31, 0.70]
Diemunsch 2007 (2)	72	541	145	526	11.8%	0.40 [0.29, 0.55]
Gan 2007	25	248	64	246	9.1%	0.32 [0.19, 0.53]
Habib 2011	7	51	19	53	4.5%	0.28 [0.11, 0.76]
**Subtotal (95% CI)**		**1183**		**1156**	**41.7%**	**0.43 [0.32, 0.59]**
Total events	171		329			
Heterogeneity: Tau^2 = 0.05; Chi^2 = 7.01, df = 4 (P = 0.14); I^2 = 43%						
Test for overall effect: Z = 5.37 (P < 0.00001)						
**1.1.2 80mg**						
de Morais 2018	5	34	13	32	3.4%	0.25 [0.08, 0.82]
Grigio 2020	5	48	6	43	3.0%	0.72 [0.20, 2.54]
Ham 2016	13	55	20	55	5.5%	0.54 [0.24, 1.24]
Kakuta 2011	13	30	27	30	2.6%	0.08 [0.02, 0.34]
Kawano 2015	1	30	3	30	1.1%	0.31 [0.03, 3.17]
Lee 2012	22	42	34	42	4.5%	0.26 [0.10, 0.69]
Lim 2013	5	28	7	24	2.9%	0.53 [0.14, 1.95]
Seon Jung 2012	0	40	17	40	0.7%	0.02 [0.00, 0.29]
Sinha 2013	2	64	9	60	2.1%	0.18 [0.04, 0.88]
Spaniolas 2020	0	41	4	42	0.7%	0.10 [0.01, 1.98]
Thanuja 2021	4	32	10	33	3.0%	0.33 [0.09, 1.19]
Yoo 2018	22	41	23	44	5.4%	1.06 [0.45, 2.48]
**Subtotal (95% CI)**		**485**		**475**	**34.8%**	**0.33 [0.20, 0.56]**
Total events	92		173			
Heterogeneity: Tau^2 = 0.34; Chi^2 = 19.78, df = 11 (P = 0.05); I^2 = 44%						
Test for overall effect: Z = 4.19 (P < 0.0001)						
**1.1.3 125mg**						
Diemunsch 2007 (1)	41	293	81	280	10.3%	0.40 [0.26, 0.61]
Diemunsch 2007 (2)	52	532	145	526	11.4%	0.28 [0.20, 0.40]
Gan 2007	12	293	64	246	0.0%	0.12 [0.06, 0.23]
Lim 2013	1	26	7	24	1.2%	0.10 [0.01, 0.86]
Seon Jung 2012	0	40	17	40	0.7%	0.02 [0.00, 0.29]
**Subtotal (95% CI)**		**891**		**870**	**23.5%**	**0.28 [0.16, 0.49]**
Total events	94		250			
Heterogeneity: Tau^2 = 0.14; Chi^2 = 6.96, df = 3 (P = 0.07); I^2 = 57%						
Test for overall effect: Z = 4.47 (P < 0.00001)						
**Total (95% CI)**		**2559**		**2501**	**100.0%**	**0.37 [0.29, 0.47]**
Total events	357		752			
Heterogeneity: Tau^2 = 0.11; Chi^2 = 36.55, df = 20 (P = 0.01); I^2 = 45%						
Test for overall effect: Z = 7.86 (P < 0.00001)						
Test for subgroup differences: Chi^2 = 2.09, df = 2 (P = 0.35), I^2 = 4.2%						

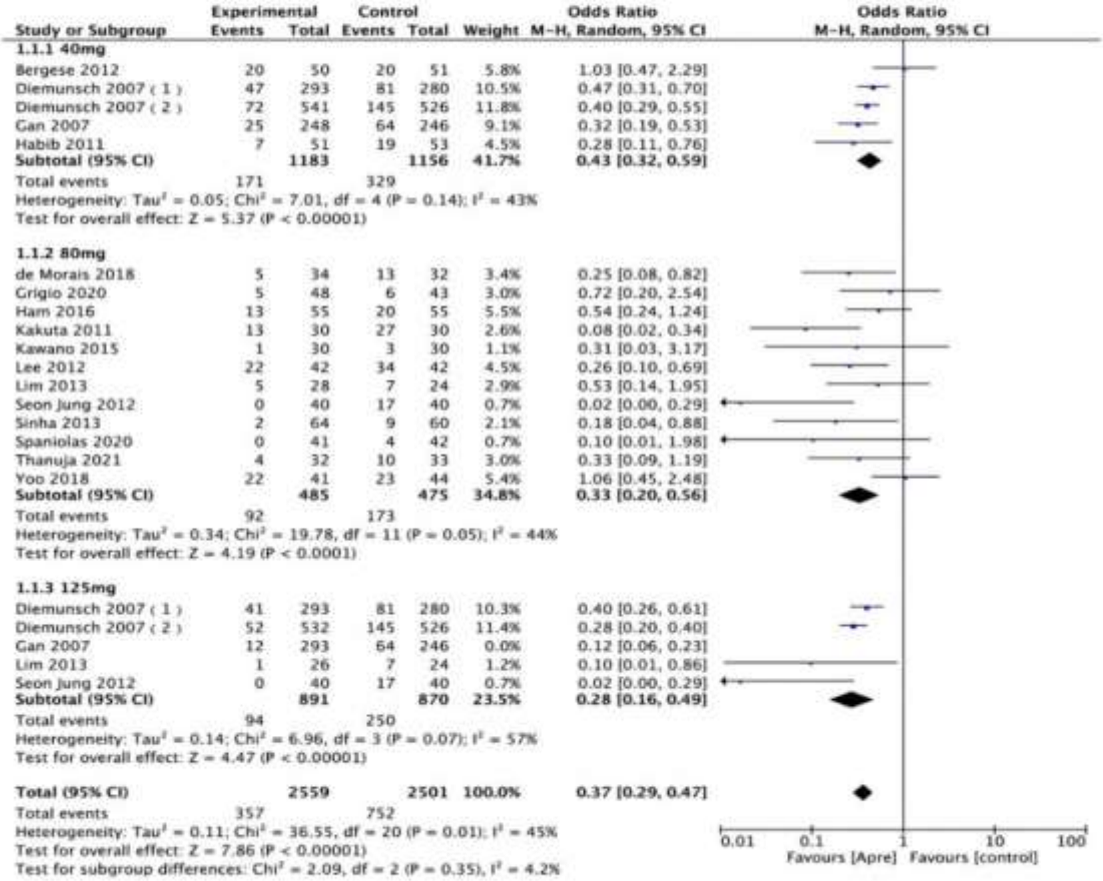

B. Incidence of vomiting after sensitivity analysis

Study or Subgroup	Experimental Events	Experimental Total	Control Events	Control Total	Weight	Odds Ratio M-H, Random, 95% CI
de Morais 2018	1	34	13	32	14.0%	0.04 [0.01, 0.37]
Grigio 2020 (1)	5	48	6	43	22.7%	0.72 [0.20, 2.54]
Ham 2016	13	55	20	55	0.0%	0.54 [0.24, 1.24]
Lee 2012	2	42	18	42	19.4%	0.07 [0.01, 0.31]
Seon Jung 2012	0	40	11	40	9.3%	0.03 [0.00, 0.56]
Thanuja 2021	3	32	9	33	20.9%	0.28 [0.07, 1.13]
Yoo 2018	1	41	6	44	13.6%	0.16 [0.02, 1.38]
**Total (95% CI)**		**237**		**234**	**100.0%**	**0.15 [0.05, 0.42]**
Total events	12		63			
Heterogeneity: Tau^2 = 0.78; Chi^2 = 10.00, df = 5 (P = 0.08); I^2 = 50%						
Test for overall effect: Z = 3.60 (P = 0.0003)						

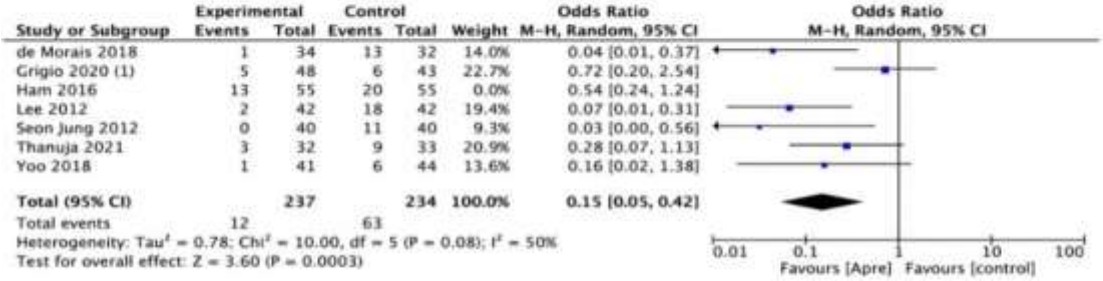

C. Incidence of nausea after sensitivity analysis

Study or Subgroup	Experimental Events	Experimental Total	Control Events	Control Total	Weight	Odds Ratio M-H, Random, 95% CI
de Morais 2018	5	34	1	34	10.2%	5.69 [0.63, 51.57]
Grigio 2020	17	48	6	48	25.2%	3.84 [1.36, 10.86]
Ham 2016	33	55	13	55	29.9%	4.85 [2.13, 11.04]
Lee 2012	21	42	2	42	16.8%	20.00 [4.27, 93.63]
Seon Jung 2012	11	40	0	40	6.7%	31.58 [1.79, 557.41]
Yoo 2018	22	41	1	41	11.2%	46.32 [5.80, 369.65]
**Total (95% CI)**		**260**		**260**	**100.0%**	**8.60 [3.84, 19.26]**
Total events	109		23			
Heterogeneity: Tau^2 = 0.39; Chi^2 = 8.59, df = 5 (P = 0.13); I^2 = 42%						
Test for overall effect: Z = 5.23 (P < 0.00001)						

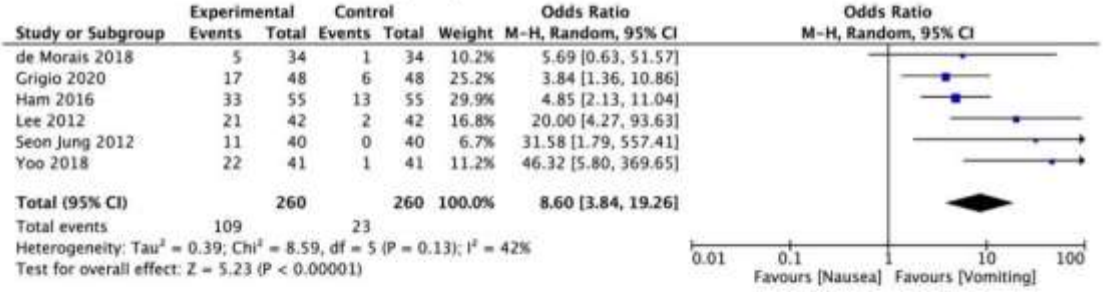

D. Complete response

Study or Subgroup	Experimental Events	Experimental Total	Control Events	Control Total	Weight	Odds Ratio M-H, Random, 95% CI
**2.1.1 40mg**						
Bergese 2012	41	50	38	51	2.6%	1.56 [0.60, 4.06]
Diemunsch 2007 (1)	188	293	154	280	12.6%	1.46 [1.05, 2.05]
Diemunsch 2007 (2)	205	541	164	526	16.2%	1.35 [1.05, 1.74]
Gan 2007	117	248	103	246	11.8%	1.24 [0.87, 1.77]
Habib 2011	14	51	21	53	3.4%	0.58 [0.25, 1.32]
**Subtotal (95% CI)**		**1183**		**1156**	**46.6%**	**1.30 [1.08, 1.57]**
Total events	565		480			
Heterogeneity: Tau² = 0.01; Chi² = 4.49, df = 4 (P = 0.34); I² = 11%						
Test for overall effect: Z = 2.76 (P = 0.006)						
**2.1.2 80mg**						
Ham 2016	21	55	9	55	3.0%	3.16 [1.29, 7.75]
Seon Jung 2012	27	64	22	60	4.3%	1.26 [0.61, 2.60]
Sinha 2013	26	40	15	40	2.9%	3.10 [1.24, 7.71]
**Subtotal (95% CI)**		**159**		**155**	**10.2%**	**2.19 [1.16, 4.12]**
Total events	74		46			
Heterogeneity: Tau² = 0.13; Chi² = 3.41, df = 2 (P = 0.18); I² = 41%						
Test for overall effect: Z = 2.43 (P = 0.02)						
**2.1.3 125mg**						
Diemunsch 2007 (1)	185	293	154	280	12.6%	1.40 [1.00, 1.96]
Diemunsch 2007 (2)	188	541	164	526	16.1%	1.18 [0.91, 1.52]
Gan 2007	103	239	103	246	11.6%	1.05 [0.73, 1.51]
Seon Jung 2012	26	40	15	40	2.9%	3.10 [1.24, 7.71]
**Subtotal (95% CI)**		**1113**		**1092**	**43.2%**	**1.28 [1.00, 1.65]**
Total events	502		436			
Heterogeneity: Tau² = 0.03; Chi² = 5.35, df = 3 (P = 0.15); I² = 44%						
Test for overall effect: Z = 1.93 (P = 0.05)						
**Total (95% CI)**		**2455**		**2403**	**100.0%**	**1.35 [1.14, 1.59]**
Total events	1141		962			
Heterogeneity: Tau² = 0.03; Chi² = 17.29, df = 11 (P = 0.10); I² = 36%						
Test for overall effect: Z = 3.56 (P = 0.0004)						
Test for subgroup differences: Chi² = 2.52, df = 2 (P = 0.28), I² = 20.5%						

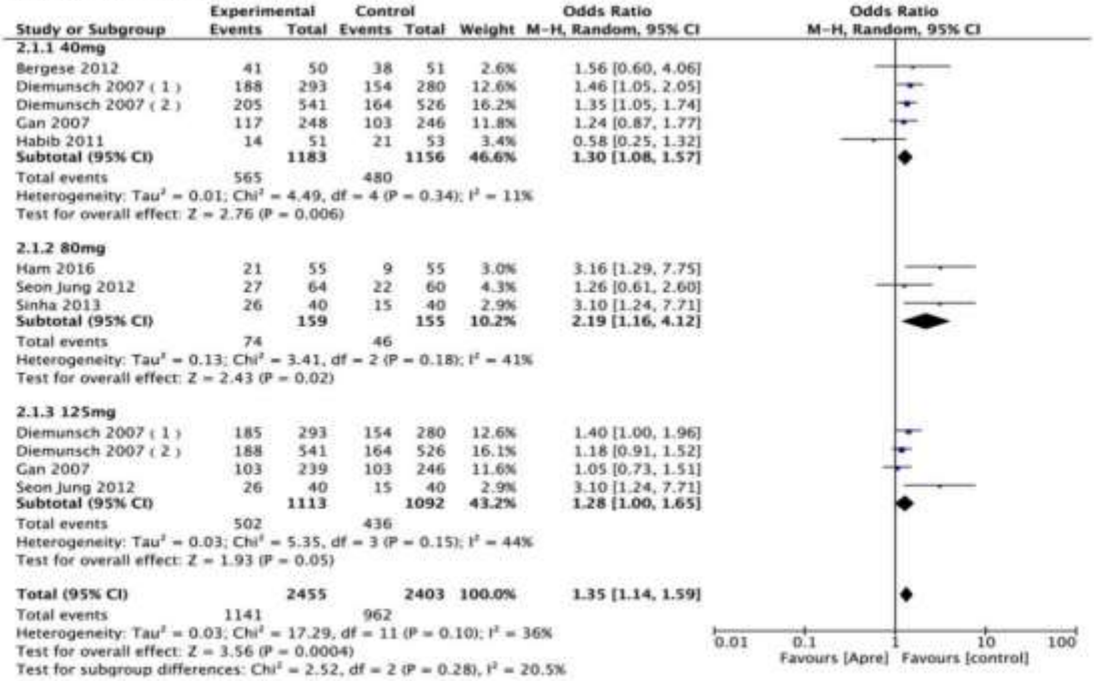

E. Use of rescue drugs

Study or Subgroup	Experimental Events	Experimental Total	Control Events	Control Total	Weight	Odds Ratio M-H, Random, 95% CI
**3.1.1 40mg**						
Diemunsch 2007 (1)	196	293	176	280	15.3%	1.19 [0.85, 1.68]
Gan 2007	112	248	113	246	14.9%	0.97 [0.68, 1.38]
Habib 2011	31	51	30	53	6.4%	1.19 [0.54, 2.60]
**Subtotal (95% CI)**		**592**		**579**	**36.6%**	**1.09 [0.86, 1.38]**
Total events	339		319			
Heterogeneity: Tau² = 0.00; Chi² = 0.74, df = 2 (P = 0.69); I² = 0%						
Test for overall effect: Z = 0.71 (P = 0.48)						
**3.1.2 80mg**						
de Morais 2018	3	32	9	32	2.4%	0.26 [0.06, 1.09]
Grigio 2020	1	48	2	43	0.9%	0.44 [0.04, 4.99]
Ham 2016	32	55	32	55	6.7%	1.00 [0.47, 2.13]
Kakuta 2011	1	30	9	30	1.1%	0.08 [0.01, 0.68]
Lee 2012	12	42	22	42	5.1%	0.36 [0.15, 0.90]
Lim 2013	2	28	6	24	1.7%	0.23 [0.04, 1.28]
Sinha 2013	27	64	26	60	7.3%	0.95 [0.47, 1.94]
Thanuja 2021	4	32	6	33	2.5%	0.64 [0.16, 2.53]
Yoo 2018	12	41	14	44	4.9%	0.89 [0.35, 2.24]
**Subtotal (95% CI)**		**372**		**363**	**32.7%**	**0.58 [0.37, 0.91]**
Total events	94		126			
Heterogeneity: Tau² = 0.13; Chi² = 11.17, df = 8 (P = 0.19); I² = 28%						
Test for overall effect: Z = 2.37 (P = 0.02)						
**3.1.3 125mg**						
Diemunsch 2007 (1)	190	293	176	280	15.3%	1.09 [0.78, 1.53]
Gan 2007	105	239	113	246	14.8%	0.92 [0.64, 1.32]
Lim 2013	0	26	6	24	0.6%	0.05 [0.00, 1.01]
**Subtotal (95% CI)**		**558**		**550**	**30.8%**	**0.94 [0.61, 1.46]**
Total events	295		295			
Heterogeneity: Tau² = 0.07; Chi² = 4.29, df = 2 (P = 0.12); I² = 53%						
Test for overall effect: Z = 0.27 (P = 0.79)						
**Total (95% CI)**		**1522**		**1492**	**100.0%**	**0.86 [0.68, 1.09]**
Total events	728		740			
Heterogeneity: Tau² = 0.06; Chi² = 22.44, df = 14 (P = 0.07); I² = 38%						
Test for overall effect: Z = 1.25 (P = 0.21)						
Test for subgroup differences: Chi² = 5.92, df = 2 (P = 0.05), I² = 66.2%						

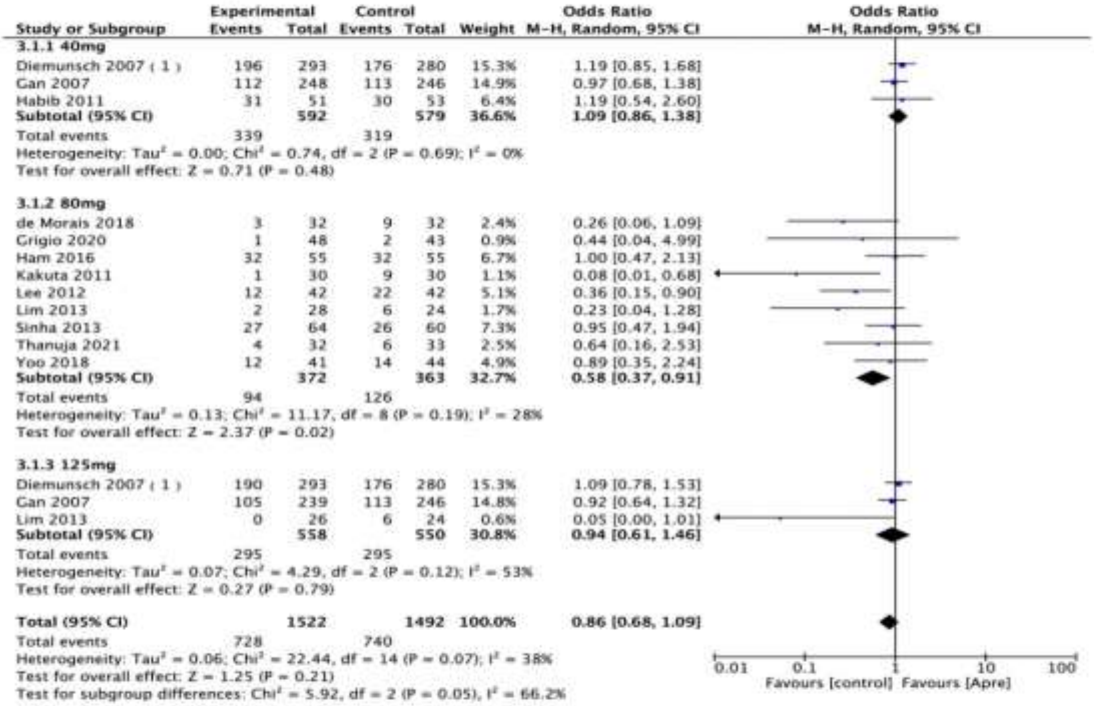

F. Analgesic

Study or Subgroup	Experimental Mean	Experimental SD	Experimental Total	Control Mean	Control SD	Control Total	Weight	Mean Difference IV, Random, 95% CI
Grigio 2020	5.35	6.8	39	3.84	4.34	34	52.8%	1.51 [-1.08, 4.10]
Kakuta 2011	3	6.1	30	7	8.1	30	47.2%	-4.00 [-7.63, -0.37]
**Total (95% CI)**			**69**			**64**	**100.0%**	**-1.09 [-6.48, 4.30]**
Heterogeneity: Tau² = 12.60; Chi² = 5.88, df = 1 (P = 0.02); I² = 83%								
Test for overall effect: Z = 0.40 (P = 0.69)								

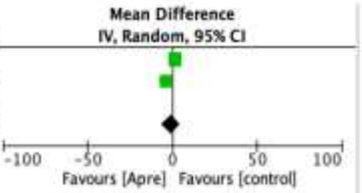

Supplement: Supplementary file 1 [file medi-102-e34385-s001.pdf]
