# Supplementary figures and images for: The efficacy of aprepitant for the prevention of postoperative nausea and vomiting: A meta-analysis
Source: Medicine (Baltimore). 2023 Jul 21;102(29):e34385. doi: 10.1097/MD.0000000000034385 (PMC10662847; doi:10.1097/MD.0000000000034385)

Figure S2. Funnel plot.

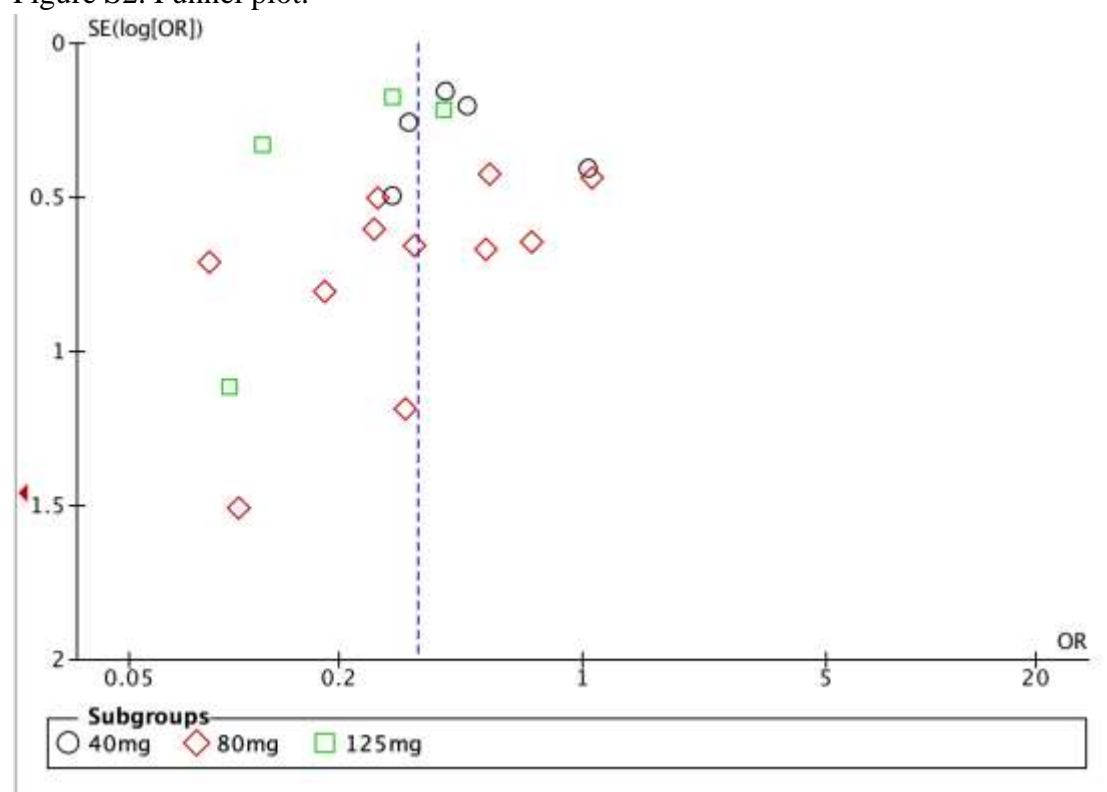

Supplement: Supplementary file 2 [file medi-102-e34385-s002.pdf]
